# On the Size Effect of Additives in Amorphous Shape Memory Polymers

**DOI:** 10.3390/ma14020327

**Published:** 2021-01-10

**Authors:** Elias M. Zirdehi, Hakan Dumlu, Gunther Eggeler, Fathollah Varnik

**Affiliations:** 1 Interdisciplinary Centre for Advanced Materials Simulation (ICAMS), Ruhr-Universität Bochum, Universitätsstr. 150, 44801 Bochum, Germany; elias.mahmoudinezhad@ruhr-uni-bochum.de; 2 Institute for Materials (IFM), Ruhr-Universität Bochum, Universitätsstr. 150, 44801 Bochum, Germany; hakan.dumlu@ruhr-uni-bochum.de (H.D.); gunther.eggeler@ruhr-uni-bochum.de (G.E.)

**Keywords:** shape memory polymers, size effect of additives, molecular dynamics simulations

## Abstract

Small additive molecules often enhance structural relaxation in polymers. We explore this effect in a thermoplastic shape memory polymer via molecular dynamics simulations. The additive-to-monomer size ratio is shown to play a key role here. While the effect of additive-concentration on the rate of shape recovery is found to be monotonic in the investigated range, a non-monotonic dependence on the size-ratio emerges at temperatures close to the glass transition. This work thus identifies the additives’ size to be a qualitatively novel parameter for controlling the recovery process in polymer-based shape memory materials.

## 1. Introduction

Shape memory polymers (SMPs) are a promising class of functional materials with interesting properties and a wide range of applications [1,2]. Some of these features such as biocompatibility [3,4,5] and large strain recovery make them better candidates particularly in biomedical field compared to metallic shape memory alloys [6,7]. The thermo-responsive shape memory effect (SME) is an intrinsic feature among almost all the polymers due to their entropic elasticity, that is, a tendency of the stretched chains to deform back towards the most probable conformation [8]. However, exploiting the entropic elasticity in order to generate a usable SME depends on the molecular architecture of polymer as well as an appropriate programming procedure [7,9].

Depending on the underlying mechanism, the shape recovery in SMPs can be triggered in different ways such as by thermal, electrical and magnetic stimuli, as well as by light, ultrasound energy, moisture and change in PH [10,11,12,13,14,15,16,17]. Crucial to shape memory effect in polymers is the change of chains’ conformation upon deformation and the tendency to recover a high entropic state during structural relaxation. These high entropic conformations have been recently studied for quiescent copolymers of different architectures at low temperatures and under poor-solvent conditions [18,19,20].

In most of the biomedical applications for SMPs, the sample shall operate and remain effective in a chemical environment [21,22,23,24,25,26]. Therefore, a question of high importance concerning the functionality of SMPs in physiological environments is how efficiently they can perform when small molecules are absorbed into their structure from the environment.

Small molecules are reported to influence morphology [27] and relaxation dynamics [28,29,30,31,32,33,34,35,36] in polymers. The glass transition temperature, $T_{g}$, of the polymers decreases upon absorbing small molecules [31]. It is reported that this effect can also lead to an antiplasticization by enhancing the local stiffness [32]. Furthermore, antiplasticizers are found to alter the nature of glass formation via enhancing the packing efficiency in polymers [28] which leads to the formation of stronger glass-forming materials [30]. In thermoplastic SMPs, the additive-effect often manifests itself in a shift of the $T_{g}$ and the onset of shape recovery at a lower temperature as compared to the pure sample [37]. This solvent-induced effect on SME has been investigated experimentally by many researchers [37,38,39,40,41,42,43,44,45,46,47,48]. A recent experimental study reported on a new strategy to tune the switching temperature through sequence-rearranged cocrystallization of copolymer blocks [49].

The shape memory behavior in polymers has also been investigated by means of molecular dynamics (MD) computer simulations [50,51,52,53,54,55,56]. In a recent MD-work [54], different models of polymers were analyzed in a shape memory cycle, highlighting the importance of temperature-dependent chain mobility. Another study [56] employed dissipative particle dynamics (DPD) to investigate phase-separation in the polymer network and individual functions of different segments in a polyurethane-based shape memory polymer. As to additive-induced shape recovery, it has been shown that plasticizing effect of additive molecules on structural relaxation in polymers leads to an enhancement of shape recovery rate [53,57,58].

In the present work, MD simulations are employed to explore additive effects on shape memory behavior with a qualitatively new perspective, showing that the size of absorbed molecules provides a new independent parameter to control the shape recovery process. Importantly, at a constant number concentration, the temperature at which significant relaxation towards the original shape takes place changes in a non-monotonic way with the additive diameter.

## 2. Model and Methods

### 2.1. Model

In this study, we use a toy model made of a sequence of strongly and weakly interacting spherical particles which we loosely name ‘hard’ and ‘soft’, arranged in a way as to mimic the sequence of hard and soft segments in ESTANE^®^. The model consists of ar block copolymers with two monomer types A and B. The connectivity of the chains’ backbone is ensured via a FENE potential [59,60],

(1) $$U_{\mathrm{FENE}}\left(r\right)=-\frac{1}{2}kR_{0}^{2}\ln\left[1-{\left(\frac{r}{R_{0}}\right)}^{2}\right],$$

where k=30 is the strength factor and $R_{0}=1.5$ is the breaking limit of covalent bonds. In addition to the FENE-potential, all particle pairs (including spherical additive molecules) interact via a Lennard-Jones (LJ) potential,

(2) $$U_{\alpha \beta }^{\mathrm{LJ}}\left(r_{\alpha \beta }\right)=4\varepsilon_{\alpha \beta }\left[{\left(\frac{\sigma_{\alpha \beta }}{r_{\alpha \beta }}\right)}^{12}-{\left(\frac{\sigma_{\alpha \beta }}{r_{\alpha \beta }}\right)}^{6}\right].$$

In Equation (2), α,β∈{A,BandS}, where the letter S stands for spherical additive particles. $r_{\alpha \beta }$ is a short hand notation for the distance between a particle, *i*, of type α and another one, *j*, of type β: $r_{\alpha \beta }=\left|{\vec{r}}_{i,\alpha }-{\vec{r}}_{j,\beta }\right|$. The LJ potential is truncated at a cutoff radius of $r_{c,\alpha \beta }=2\times 2^{1/6}\sigma_{\alpha \beta }$. The monomer diameter is set to $\sigma_{\mathrm{AA}}=\sigma_{\mathrm{BB}}\equiv 1$ and serves as unit of length. For α≠β, the parameter $\sigma_{\alpha \beta }$ is chosen as the arithmetic mean, $\sigma_{\alpha \beta }=0.5\left(\sigma_{\alpha \alpha }+\sigma_{\beta \beta }\right)$.

**Figure 1:**
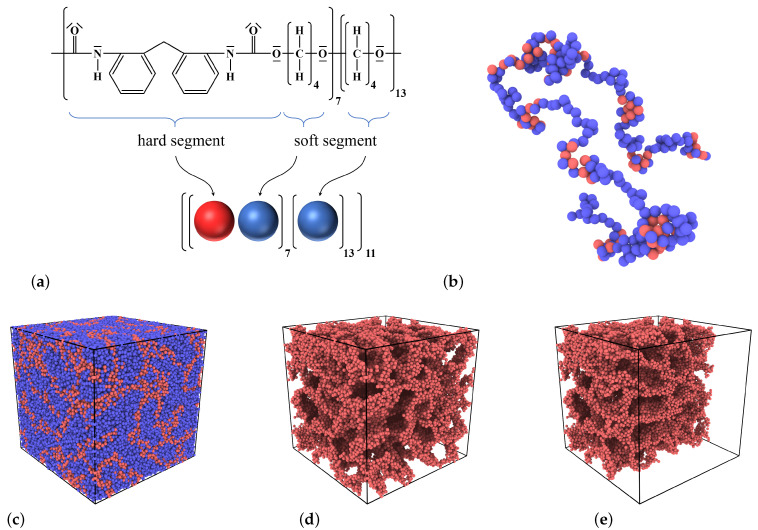
(**a**) Molecular structure of ESTANE^®^, consisting of strongly and weakly interacting (‘hard’ and ‘soft’) segments as indicated (Courtesy of Axel Marquardt [58]). In our simplified model, each hard segment is represented as a type A (red) particle and each soft segment by a type B (blue) sphere. The arrangement and number of A and B particles follows the sequence of hard and soft segments in the experimental sample. As a result, each bead-spring chain consists of 77 ‘hard’ and 220 ‘soft’ segments, making a total of $N_{p}=297$ monomers per chain. (**b**) A snapshot of a single chain from our simulations. (**c**) Snapshot of the simulation box containing 400 chains which shows a heterogeneous distribution of hard (red) and soft (blue) segments. (**d**) The same data as in (**c**) but hiding the soft segments. (**e**) The same data as in (**d**) but keeping only half of the simulation box. Panels (**d**,**e**) serve to highlight the phase separation between hard and soft segments on the nano-scale. The snapshots are taken from a pure sample at T=0.23.

As mentioned above, A and B monomers follow the same sequence as the hard and soft segments in ESTANE^®^ (Figure 1a,b). Using the LJ-interaction parameter of AA-particle pairs as unit of energy, $\varepsilon_{\mathrm{AA}}=1$, the BB-interaction strength is set to $\varepsilon_{\mathrm{BB}}=\varepsilon_{\mathrm{AA}}/2=0.5$. The corresponding cross term of interaction energy is chosen as $\varepsilon_{\mathrm{AB}}=0.3\varepsilon_{\mathrm{AA}}=0.3$. The justification for this choice of interaction parameters is that they lead to a phase separation between hard and soft segments on the nano-scale (Figure 1c–e), and thus reproduce an important feature—which is well-known from experiments [61,62,63]—at least on a qualitative level.

We also remark that bond-angle and torsional potentials are not included in the present model. Results discussed in this manuscript thus correspond to a generic flexible co-polymer model without reference to a specific chemistry.

The phase separation of hard and soft regions is a slow process, whose simulation requires advanced techniques. Here, we equilibrate the system using an approach proposed by Parker and Rottler [64], where the excluded-volume LJ-interaction is replaced by a soft potential. A main feature of this approach is that the chains become crossable and exhibit a Rouse-like dynamics which leads to a considerably faster relaxation.

Since the thus obtained dynamics is a biased one, we use this method only for the sample preparation step (from a random initial configuration towards a nanostructured sample). All the simulations of the shape memory cycle are performed using the full interaction model described through Equations (1) and (2). If additive particles are included, the energy scales pertaining to the corresponding spherical particles are all set to that of the soft monomers ($\varepsilon_{\mathrm{SS}}=\varepsilon_{\mathrm{AS}}=\varepsilon_{\mathrm{BS}}=\varepsilon_{\mathrm{BB}}$). The employed parameters of LJ potential for different components are summarized in Table 1. The mass of an additive particle is set to be equal to that of the monomers, $m_{S}=m_{A}=m_{B}=1$. Temperature is measured in units of $\varepsilon_{\mathrm{AA}}/k_{B}$ with the Boltzmann constant $k_{B}$ (≡1). All other quantities are given as a combination of the above described units. The unit of time, for example, is given by $\tau_{\mathrm{LJ}}={\left(m\sigma_{\mathrm{AA}}^{2}/\varepsilon_{\mathrm{AA}}\right)}^{1/2}$ and that of pressure is $\varepsilon_{\mathrm{AA}}/\sigma_{\mathrm{AA}}^{3}$.

All quantities addressed here are expressed in this set of reduced LJ units. The equations of motion are integrated using the Velocity-Verlet algorithm with a time step of δt=0.005 for the preparation of initial configurations and δt=0.003 for the actual simulations of shape memory behavior. Unless otherwise stated, all the simulations of the SMP-behavior are conducted at a constant pressure of p=0. For the simulation software, we use the open source molecular dynamics simulator LAMMPS [65]. All the 3D snapshots of particle configurations in this work are prepared by the visualization software OVITO [66].

### 2.2. Sample Preparation

**Figure 2:**
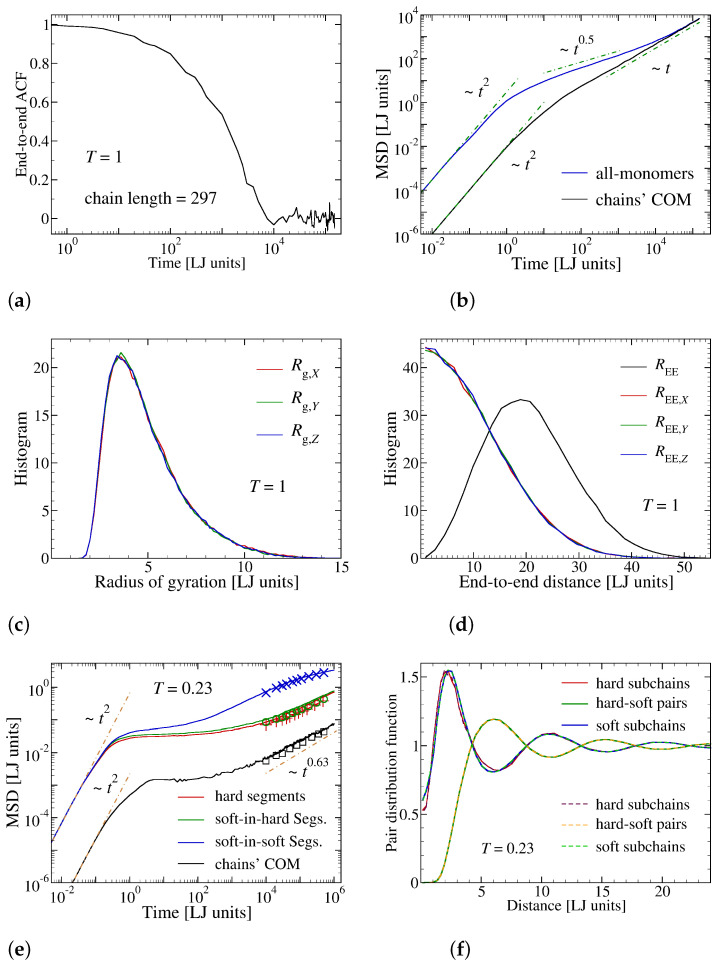
(**a**) Auto-correlation function of end-to-end vector during the initial equilibration step via soft potential approach of Parker and Rottler [64]. (**b**) Mean-squared displacement for all monomers and chains’ center of mass (COM) during the same stage as in (**a**). The short time ballistic (∼$t^{2}$) and long time diffusive (∼*t*) regimes are also highlighted. The histogram for projections of (**c**) gyration tensor and (**d**) end-to-end vector along different directions show perfect isotropy. (**e**,**f**) Dynamic and static properties of the model after replacing the soft potential by the LJ-excluded-volume interactions. (**e**) Mean-squared displacements of different components plotted for the full model in the absence of additive at T=0.23 (<$T_{g}\approx 0.25$, see Figure 3). The emergence of a quasi-Rouse-like dynamics, is visible (∼$t^{0.63}$). The symbols show dynamic measurements using $t_{0}=10^{5}\tau_{\mathrm{LJ}}$ (note that MSD are plotted versus $t-t_{0}$). The observed difference in the dynamics for $t_{0}=0$ (lines) and $t_{0}=10^{5}\tau_{\mathrm{LJ}}$ (Symbols) is a signature of an ongoing aging. This effect is, however, weak and can be neglected as compared to the effects of temperature and additive molecules. (**f**) Pair distribution function for hard and soft subchains, averaged over a first time interval of $t\in [0,10^{5}\tau_{\mathrm{LJ}}]$ (solid lines) and a second one $t\in [10^{5}\tau_{\mathrm{LJ}},2\times 10^{5}\tau_{\mathrm{LJ}}]$ (dashed lines), highlighting that the structure is hardly changed during aging. An anti-correlation is visible between the self and cross terms. This can be regarded as a consequence of the phase separation between the regions of hard and soft segments (Figure 1c–e).

For the preparation of initial configurations, first, a regular arrangement of the chains is simulated within a constant volume and at a fixed temperature (NVT-ensemble) for a duration of 150,000 $\tau_{\mathrm{LJ}}$ at a monomer number density of $\rho_{0}$ = 0.93 $\sigma_{\mathrm{AA}}^{-3}$ and temperature $T=1\;\varepsilon_{\mathrm{AA}}/k_{B}$ employing the above-mentioned soft repulsive potential [64]. This allows a considerably faster equilibration of our long-chain polymeric system since entanglements are avoided and the monomers follow a Rouse-like dynamics. The mean-squared displacements and end-to-end autocorrelation function plotted in Figure 2a–d demonstrate that the soft potential version of the system is completely equilibrated. Then the soft potential is reversed to the full LJ model with excluded-volume effect following the procedure explained in Reference [64]. Finally, the sample is cooled down to T=0.35 with a cooling rate of $\dot{T}=10^{-4}$ using NpT-ensemble at zero pressure.

The polymer-additive mixture for additives of $\sigma_{\mathrm{SS}}=0.5$ is prepared by inserting, one after another in regular time intervals, spherical particles into the polymer matrix, making use of the soft potential $U_{\mathrm{soft}}\left(r_{\alpha S}\right)=\varepsilon_{\alpha S}\left[1+\cos\left(\pi r_{\alpha S}/\sigma_{\alpha S}\right)\right]$, with $\varepsilon_{\alpha S}=0.5$ and $\sigma_{\alpha S}=0.5\left(\sigma_{\alpha \alpha }+\sigma_{\mathrm{SS}}\right)$, where α∈{A,B}. The number concentration is defined via $\phi_{s}=N_{s}/\left(N_{s}+N_{p}\right)$, with $N_{s}$ and $N_{p}$ being the number of additives and monomers. Particle insertion is performed during a time interval of $1500\;\tau_{\mathrm{LJ}}$ at T=0.35 in NpT-ensemble at zero pressure. Then, the sample is simulated for $500\;\tau_{\mathrm{LJ}}$ which is sufficient for the additives to travel a relatively large distance of 20 particle diameters thanks to their soft interaction (cosine potential) with the surroundings. By surveying concentration profiles along different spatial directions and also within various planes, we have verified that additives reach a uniform distribution before switching on the full LJ-potential (for brevity, data are not shown). After these steps, the energy parameter of the soft potential is gradually increased to a high value of $\varepsilon_{\alpha S}=30$ during a time interval of $100\;\tau_{\mathrm{LJ}}$. This reduction in softness of interactions serves to prevent the additives from overlapping with the surrounding particles and is necessary for avoiding high energy collisions when the LJ is switched on. Subsequently, the cosine potential is replaced by the LJ potential and the system is simulated for a time span of $9000\;\tau_{\mathrm{LJ}}$. To generate mixtures with additives of other diameters (e.g., $\sigma_{\mathrm{SS}}=0.3$ and 0.8), we do not repeat the above procedure but use the $\sigma_{\mathrm{SS}}=0.5$–configurations as the starting point and gradually change, at a constant pressure of p=0, the additive diameter $\sigma_{\mathrm{SS}}$ from 0.5 to the desired value.

The thus obtained samples are simulated for another 10,000 $\tau_{\mathrm{LJ}}$ in NpT-ensemble at zero pressure. This procedure, of course, does not lead to a perfectly equilibrated configuration of the full model. Rather, it slows down the structural relaxation and leads to a plateau in mean square displacements, signaling temporal arrest in the nearest neighbor cage (see, e.g., Figure 2e for a pure sample quenched further to T=0.23 at $\dot{T}=10^{-4}$). The structural data further underlines the disordered character of the system (Figure 2f). As a crucial aspect, we make sure that the duration of temporal arrest for all the samples prepared this way is beyond the time relevant for the main part of strain recovery, which is ≤1000 $\tau_{\mathrm{LJ}}$. This way, the shape memory effect is not deteriorated by structural relaxation.

### 2.3. Simulating the Shape Memory Effect

The samples obtained in the previous section are used as initial configurations for studying the shape memory effect via the following procedure (see Figure 4 for an illustration). First, the simulation box is stretched along the *z*-direction with a constant engineering strain rate (${\dot{\epsilon }}_{\|}=10^{-3}$), defined via $\epsilon_{\|}=dL_{z}/L_{z}\left(t=0\right)$, at a temperature above the glass transition point ($T_{\mathrm{deform}}>T_{g}$) of the sample under investigation.

**Figure 3:**
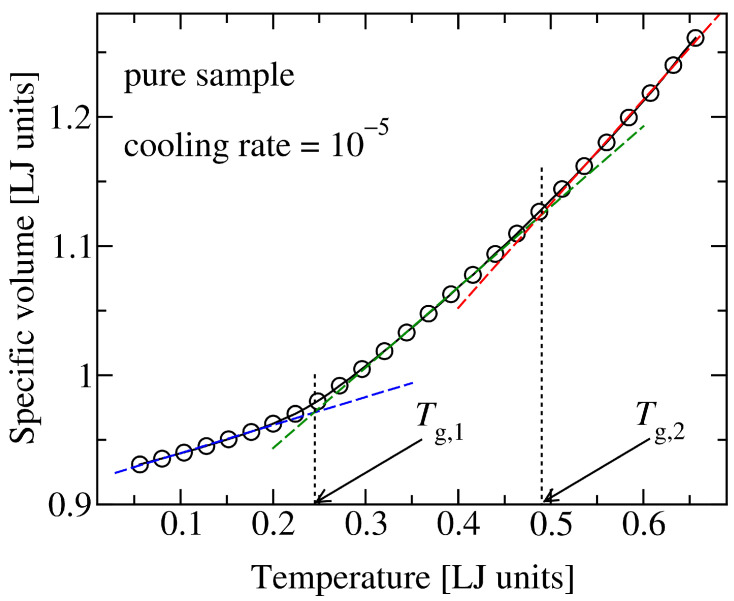
Specific volume, $\nu =V/N_{p}$, versus temperature at a fixed pressure of p=0 for a constant cooling rate of $\dot{T}=10^{-5}$. Bending of the curve signals glass transition. Here, a distinct kink is identified at low temperatures (T∈[0.2,0.3]). The intersection of linear segments determines the corresponding glass transition temperature (here $T_{g,1}\approx 0.25$). A close scrutiny of the data at higher temperatures (T>0.4) reveals a further, albeit much weaker change of slope. The associated glass transition is more difficult to determine from simulated data. Here we estimate a value of $T_{g,2}\approx 0.49$. All quantities are given in reduced Lennard-Jones units.

**Figure 4:**
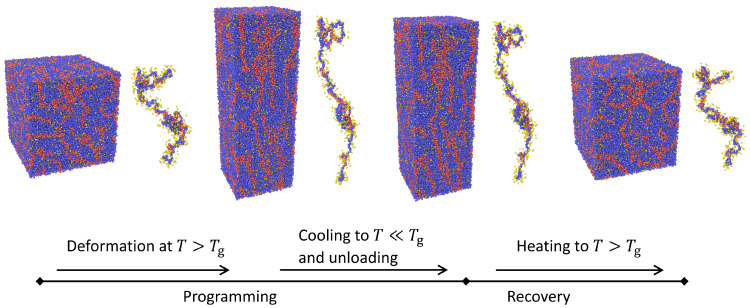
A shape memory cycle for the present bead-spring copolymer model. The sample is first deformed at a temperature above its glass transition, $T_{g}$. Then it is cooled down to a temperature below $T_{g}$ followed by unloading. The sample first deforms back slightly due to its energetic elasticity but the major part of deformation is stored as it is connected to entropic elasticity, the latter becoming active at temperatures above $T_{g}$. Therefore, shape recovery takes place only if the sample is heated to a temperature $T>T_{g}$.

It is noteworthy that, due to the presence of two distinct domains with different self-interactions, that is, hard and soft segments, the model exhibits two glass transition temperatures (Figure 3). In a simplified view, the higher $T_{g}$ can be assigned to a transition from a rubbery state to a partly glassy state, where hard segments are essentially immobile but soft segments keep significant mobility. As a consequence, shape memory effect does not occur at temperatures around this high $T_{g}$. The lower $T_{g}$, on the other hand, represents a temperature below which both hard and soft domains have negligible dynamics (are temporally arrested) within the deformation time scale. Therefore, we performed all our simulations at temperatures around and below the lower $T_{g}$. Since the higher $T_{g}$ plays no role throughout this work, we refer to the lower value of $T_{g}\approx 0.25$ as the glass transition point of the pure polymer system. Similarly, glass transition temperatures of polymer-additive mixtures refer to the lower value of the two $T_{g}$s.

During deformation, the two dimensions transverse to the direction of deformation, $L_{x}$ and $L_{y}$, are independently coupled to the Nosé-Hover barostat at zero pressure ($p_{⊥}=p_{xx}=p_{yy}=0$) and can vary in response to internal forces. Once a maximum strain of $\epsilon_{\|}^{\max}=100\%$ is reached, the sample is cooled at fixed strain to a temperature of T=0.1 with a cooling rate of ${\dot{T}}_{\mathrm{cooling}}=10^{-2}$. Applying such a fast cooling helps to freeze chain conformations in the elongated state and thus minimize the undesired effects which would arise from structural relaxation. At the end of this deep and fast cooling process, the size of the simulation box along the direction of deformation, $L_{z}$, is coupled to a barostat at $p_{\|}=p_{zz}=0$, while the lateral dimensions ($L_{x}$ and $L_{y}$) remain independently under stress-free conditions. This leads to the release of the stress and is, therefore, referred to as the unloading stage. The above discussed temporal arrest ensures that, in the absence of external load, the system still remains in the elongated state (with the exception of a small spring-back effect due to ordinary elasticity). From the beginning of deformation process up to this point is often referred to as the ‘programming’ step. Shape recovery process is triggered via heating to a temperature above the sample’s glass transition point, $T_{\mathrm{recovery}}>T_{g}$, where structural relaxation becomes sufficiently fast to activate entropic forces, which drive the shape recovery process in polymers. For all the simulations, this heating is applied at a relatively high rate (${\dot{T}}_{\mathrm{heating}}=10^{-2}$) to suppress effects of relaxation processes, which start to become active as temperature increases.

## 3. Results and Discussion

In this section, the following findings shall be demonstrated: (1) The simple copolymer model presented above is capable of entropy-driven shape memory behavior, (2) adding small molecules to the sample plays a role similar to increasing temperature with regard to shape recovery and (3) the temperature, at which significant shape recovery takes place, first decreases for smaller additive particles but then increases as the particle size falls below roughly half a monomer diameter.

### 3.1. Effect of Additive Concentration on Shape Recovery

A way to highlight the entropic effect in shape memory polymers is to monitor the chain conformation. For this purpose, we survey the dynamics of the so-called gyration tensor of a chain, defined as $S_{\alpha \beta }=\frac{1}{N_{p}}\sum_{i=1}^{N_{p}}\left(r_{i,\alpha }-R_{\mathrm{cm},\alpha }\right)\left(r_{i,\beta }-R_{\mathrm{cm},\beta }\right)$. Here, Greek letters denote spacial directions, α,β∈{x,y,z}, $N_{p}$ is the number of monomers (or segments) in a single chain (including both hard and soft segments) and $R_{\mathrm{cm},\alpha }=\frac{1}{N_{p}}\sum_{i=1}^{N_{p}}r_{i,\alpha }$ is the αth-component of chain’s center of mass position. The symbol $\langle \cdots \rangle$ stands for statistical average.

**Figure 5:**
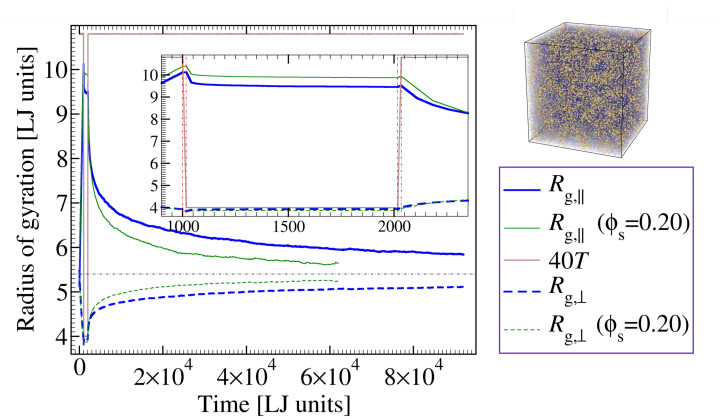
(Color online) Gyration tensor of polymer chains along the directions parallel (‖, solid lines) and perpendicular (⊥, dashed lines) to the direction of deformation. Thick blue lines correspond to a pure sample and green lines to a probe containing 20% additives with $\sigma_{\mathrm{SS}}=0.8$. End of deformation is marked by the leftmost vertical line. After the cooling stage (between the 1st and 2nd vertical lines), $L_{z}$ is coupled to a barostat with $p_{zz}=0$. This leads to a small partial recovery, followed by a plateau (best visible in the inset) indicative of temporal arrest (shape fixation). As temperature raises above $T_{g}$, chains become sufficiently mobile to acquire isotropic conformation and the probe relaxes to its original shape. The third and fourth vertical lines in the inset mark the beginning and end of the heating process. For better visibility, *T* is multiplied by a factor of 40. The upper right image is a snapshot of an undeformed configuration at T=0.35 with 20% additive particles (yellow). Monomers (blue) are made semi-transparent.

A first set of results obtained within the present model is shown in Figure 5, for the cases of a pure sample and a polymer-additive mixture. In the both cases investigated, deformation starts at a temperature slightly above the glass transition point ($T_{\mathrm{deform}}\left(\phi_{s}=0\right)=0.27>T_{g}\left(\phi_{s}=0\right)\approx 0.25$ and $T_{\mathrm{deform}}\left(\phi_{s}=0.20\right)=0.27>T_{g}\left(\phi_{s}=0.20\right)\approx 0.23$), where the system is soft enough to prevent it from crazing [67,68]. The plot depicts projections of the chains’ gyration tensor onto the directions parallel and perpendicular to deformation, $R_{g,⊥}\equiv \sqrt{\frac{1}{2}\langle S_{xx}+S_{yy}\rangle }$ and $R_{g,\|}\equiv \sqrt{\langle S_{zz}\rangle }$. One can clearly see from Figure 5 the increase in anisotropy of chain conformation during the externally imposed deformation (see data for $t<1000\;\tau_{\mathrm{LJ}}$). As the load is removed, the chain conformation still remains in this out-of-equilibrium state as long as temperature is kept sufficiently low ($T=0.1\ll T_{g}$). Upon heating to a temperature above $T_{g}$ ($T_{\mathrm{recovery}}=0.27$), however, $R_{g,\|}$ starts to decrease while at the same time $R_{g,⊥}$ increases, both converging towards one another with time, thus signaling the evolution of chain conformation towards an isotropic distribution. It is, in fact, this spontaneous process of regaining isotropic conformation, which drives shape recovery. Importantly, Figure 5 provides evidence that, within a shift in recovery temperature, the same entropy driven scenario is prevalent both in a pure polymer sample and in a probe containing additive molecules.

**Figure 6:**
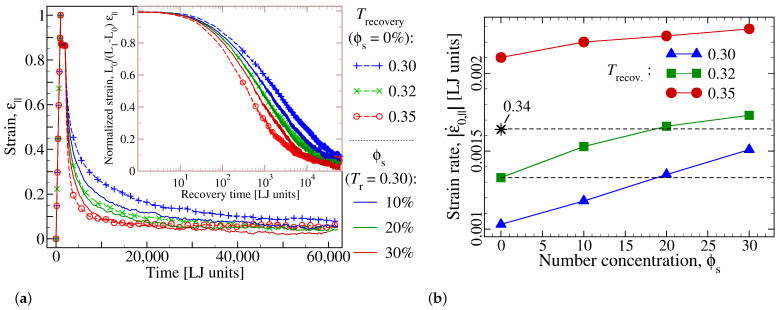
(**a**) Strain versus time for a pure sample ($\phi_{s}=0$) at different recovery temperatures and for polymer-additive mixtures at a fixed $T_{\mathrm{recovery}}=0.30$ for three choices of concentration, $\phi_{s}$ (see curve legends) and for additives of size $\sigma_{\mathrm{SS}}=0.5$. The presence of additive molecules leads to a higher recovery rate and a lower residual strain. This observation is highlighted further by showing in the inset the same data in log-linear scale. (**b**) The initial strain recovery rate, ${\dot{\epsilon }}_{0,\|}$, versus additive concentration, $\phi_{s}$, for different recovery temperatures as indicated. The concentration-dependence is more pronounced at lower $T_{\mathrm{recovery}}$. A recovery rate observed in a pure sample at a given temperature can be realized at a lower *T* if additives are added to the polymer matrix. Two such examples are highlighted by horizontal dashed lines, $\left(T=0.34,\phi_{s}=0\right)\to \left(T=0.32,\phi_{s}\approx 0.19\right)$ and $\left(T=0.32,\phi_{s}=0\right)\to \left(T=0.30,\phi_{s}\approx 0.19\right)$. The overlap of the green curves in panel (**a**) is in line with this expectation.

In view of the close connection between shape recovery and chains’ mobility, the upper temperature to which the sample is reheated plays a major role for the recovery rate. To address the influence of this ‘recovery temperature’, $T_{\mathrm{recovery}}$, statistically equivalent copies are prepared in exactly the same way until the end of programming step. Then, temperature is raised to different $T_{\mathrm{recovery}}$-values, all above $T_{g}$. As shown in Figure 6a, rate of shape recovery is enhanced at higher $T_{\mathrm{recovery}}$, consistent with the idea that kinetics of chain relaxation is faster at a higher temperature. Similarly, since adding small molecules also affects the chains’ relaxation dynamics, results for a number of additive concentrations are shown in the same plot. Similar to a raise in $T_{\mathrm{recovery}}$, one observes an enhancement of recovery rate upon increasing the number concentration of additive molecules.

A closer scrutiny of the data shown in Figure 6a reveals that recovery profile of a pure sample at $T_{\mathrm{recovery}}=0.32$ is quite similar to that a polymer-additive blend at a concentration of $\phi_{s}=20\%$ but a lower temperature of $T_{\mathrm{recovery}}=0.30$. Clearly, the initial strain relaxation is very similar in both cases and extends down to a strain value of roughly 0.2, below which slight deviations become visible. If one is interested in the first 70–80% of strain recovery, additive essentially play the role of a higher temperature. This interpretation is confirmed further by observing that a larger *T*-reduction (from $T_{\mathrm{recovery}}=0.34$ to $T_{\mathrm{recovery}}=0.30$) is possible if a higher additive concentration ($\phi_{s}=0.30$) is used. These findings are in qualitative agreement with experimental report on the effects of moisture and organic solvents on shape recovery in SMPs [38,39,40,42,58,69].

A more quantitative analysis of this observation is provided in Figure 6b, where the initial recovery rate—defined via ${\dot{\epsilon }}_{0,\|}=\left[\epsilon_{\|}\left(t\right)-\epsilon_{\|}\left(t_{0}\right)\right]/\left(t-t_{0}\right)$ with $t-t_{0}=150\tau_{\mathrm{LJ}}$— is depicted for a number of concentrations and three different values of $T_{\mathrm{recovery}}$. The plot also highlights the above mentioned idea that the same recovery rate can be realized by various combinations of temperature and additive-concentration.

An important consequence of this observation is that additive molecules provide an independent way to trigger the shape recovery process, since one can keep *T* constant but allow for penetration of additive molecules into the sample. While this finding is in line with previous studies [36,47,53], the effect of additive-to-monomer size ratio on shape recovery process has not yet been explored in the literature. The next subsection is devoted to this novel aspect.

### 3.2. Effect of the Additive Size

It has been shown above that the plasticizing effect of small molecules, which penetrate into the polymer sample, remains intact in a shape memory cycle to the extent that, additives’ concentration can be used as a new control parameter to influence the shape recovery process. Here, we demonstrate a further and qualitatively new way of using additive molecules to control the shape memory effect. This second independent route is related to the molecules’ size relative to a monomer unit.

To focus on this new effect, we follow the same procedure as described on page 6 and prepare polymer-additive mixtures for different sizes of additive particles. In all the samples investigated below, the number concentration is kept constant, $\phi_{s}=0.20$. First, a series of simulations are performed for different probes using exactly the same shape memory protocol with $T_{\mathrm{deform}}=T_{\mathrm{recovery}}=0.23$. This temperature is slightly below the glass transition point of the pure sample ($T_{g}\left(\phi_{s}=0\right)\approx 0.25$).

**Figure 7:**
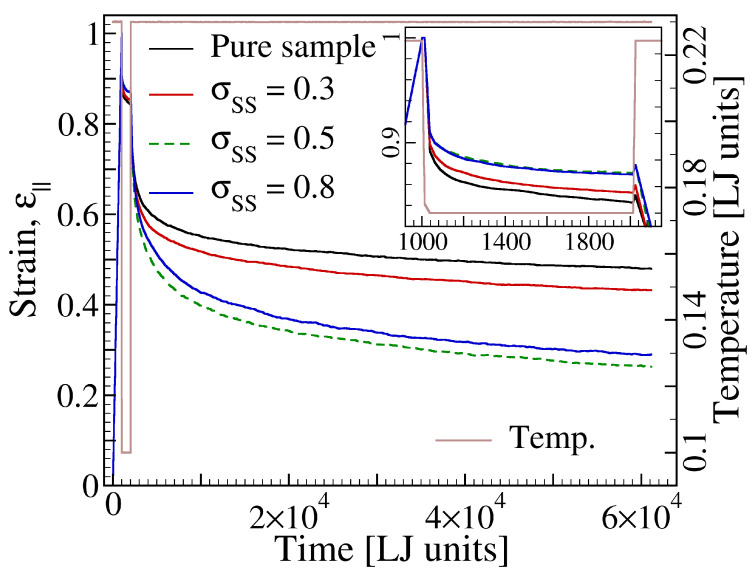
Additive size effect on shape recovery. The plot shows strain versus time in a shape memory cycle for a pure polymer and polymer-additive mixtures at $\phi_{s}=0.20$ for three choices of additive’s diameter, $\sigma_{\mathrm{SS}}$, as indicated. The recovery temperature is $T_{\mathrm{recovery}}=0.23$. A close scrutiny of the data reveals a non-monotonic dependence of the shape recovery process on particle size, the largest recovery occurring for the sample with $\sigma_{\mathrm{SS}}=0.5$.

Strain profiles obtained from these studies are shown in Figure 7. As expected, under load, the parallel component of strain, $\epsilon_{\|}$, evolves in identical way for all the cases shown. As the load is released, strain follows different paths depending on the size of molecules added to the polymer. A close survey of the data reveals that the presence of additive molecules reduces the early elastic response, which occurs right after load removal. This effect becomes stronger between additive sizes $\sigma_{\mathrm{SS}}=0.3$ and $\sigma_{\mathrm{SS}}=0.5$ but then seems to saturate with further increase of $\sigma_{\mathrm{SS}}$.

**Figure 8:**
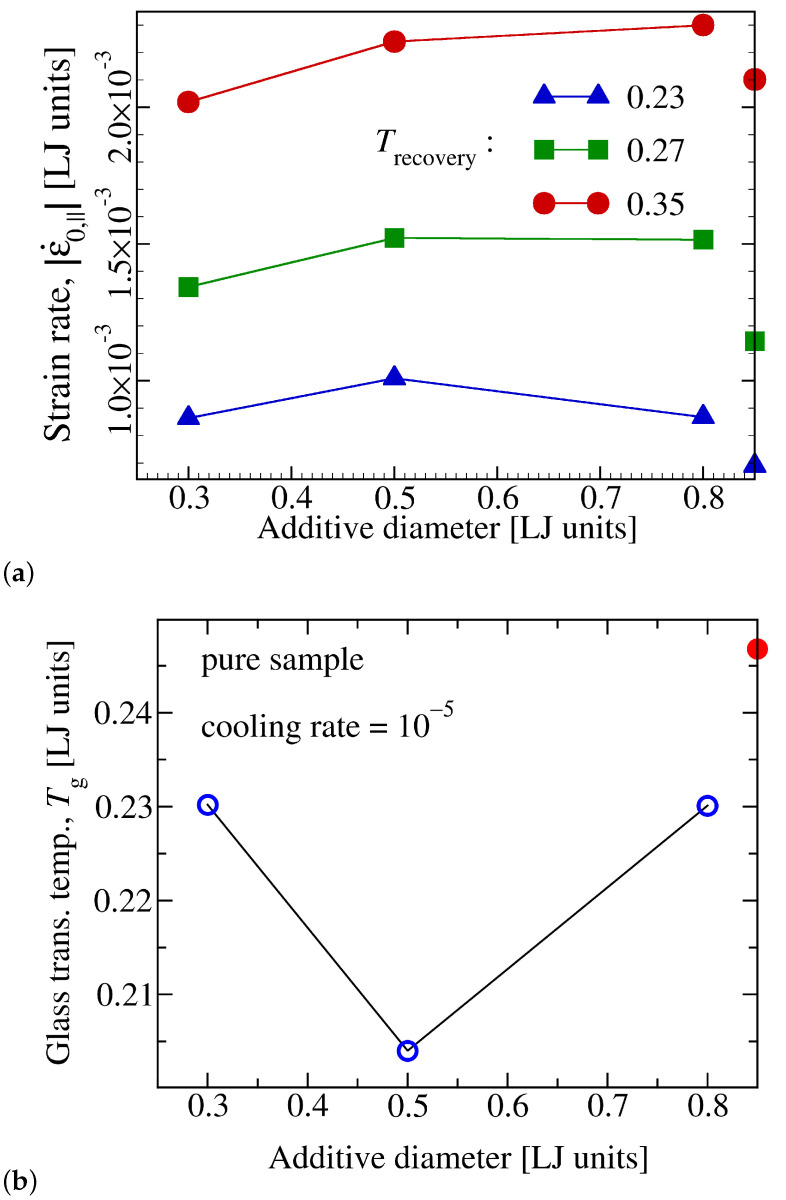
(**a**) Initial shape recovery rate versus $\sigma_{\mathrm{SS}}$ for three values of recovery temperature as indicated and for a constant number concentration of $\phi_{s}=0.20$. At the highest temperature shown here ($T_{\mathrm{recovery}}=0.35$), the rate of strain recovery is a monotonic function of $\sigma_{\mathrm{SS}}$ but a non-monotonic trend develops for lower $T_{\mathrm{recovery}}$ and becomes distinguishable at a temperature of $T_{\mathrm{recovery}}=0.23<T_{g}\left(\phi_{s}=0\right)\approx 0.25$. Filled symbols on the right vertical axis give the recovery rate of the pure sample at the corresponding $T_{\mathrm{recovery}}$. (**b**) The glass transition temperature, $T_{g}$, obtained from cooling simulations (Figure 3) versus additive-diameter. In agreement with low-*T* data in panel (**a**), a non-monotonic effect is clearly visible here. All quantities are given in Lennard-Jones units.

However, the most prominent effect of molecular size shows itself in the early stage of shape recovery, where a non-monotonic dependence of recovery rate on $\sigma_{\mathrm{SS}}$ becomes visible (Figure 8a). Noteworthy, this non-monotonic effect is a specific feature of low temperature response, as the rate of strain decrease is a monotonic function of $\sigma_{\mathrm{SS}}$ at higher temperatures. It is, therefore, intriguing to check whether this effect extrapolates to lower temperatures to the point of being observed in the glass transition temperature. To examine this idea, we have determined $T_{g}$ via cooling simulations at a constant additive concentration but different additive-diameters. These simulations provide evidence that the glass transition temperature depends on the additive diameter in a non-monotonic way (Figure 8b).

## 4. Summary and Outlook

In this work, a simple bead-spring type copolymer model is used to study shape memory effect in AB-copolymers. The model is inspired by ESTANE^®^, but has no specific chemistry. Rather, A and B monomers are arranged in the same sequence as the so-called hard and soft segments occur in the real material. Regardless of this simplification, the model captures well the shape memory behavior. This fact is traced back to the central role of entropic elasticity and the possibility to freeze non-equilibrium (elongated) chain conformations via cooling below the glass transition.

Using this model, the effect of added spherical particles on shape recovery process is investigated and is shown to be qualitatively similar to that of temperature. Plasticizing effects of additives on shape memory polymers are also reported from experiments [38,39,40,42,58,69]. The fact that a simple copolymer model reproduces such interesting effects hints towards the generic character of the phenomenon, encoded in frozen entropic elasticity.

In addition to these findings, it is shown that at temperatures close to the glass transition point, the rate of shape recovery first increases but then decreases again for progressively larger additive particles. This non-monotonic effect is shown to be also present in the dependence of the glass transition temperature on additive diameter.

Non-monotonic effects result from competing trends which develop in opposite ways as a control parameter, for example, temperature, is varied [70,71,72]. Recently, a mechanism was proposed to rationalize such non-monotonic behavior based on the competition between higher mobility of smaller additive molecules versus stronger additive-host coupling strength for larger ones [73]. A key aspect here is the strong sensitivity of structural relaxation dynamics to tiny changes of local particle packing in the proximity of $T_{g}$ [74]. This issue is also highlighted in recent theoretical developments [75,76] and experiments [77,78] on binary colloidal mixtures—where packing effects are most prominent. The connection between local packing and structural relaxation dynamics is, however, a subtle one. Importantly, we do not observe a non-monotonic variation of free volume with additive-size, which could serve as a direct cause of the observed phenomenon. For more details, the interested reader is referred to the related literature [73,74,75,76,77,78].

Regarding further theoretical work, the non-monotonic size effect shown in this work deserves special notice, as it occurs in polymers’ response to large deformation strains. This is a highly non-trivial finding and motivates new theoretical work. In view of the evidences obtained from the studies of various quiescent systems, it seems promising if one could develop a theory for polymers-additive systems which accounts both for local packing features and at the same time considers large deformation strains.

It must also be emphasized that the reported size dependence is a subtle effect and needs being examined in the case of a specific chemistry. It would be very interesting to check this issue using more realistic atomistic models. This would also open the way to investigate molecular coupling mechanisms between additive molecules and polymer and thus better understand how small molecules influence the structure and dynamics of the polymer matrix and hence the shape memory effect.

Our results also motivate new experiments with a focus on particle size effects. A possibility here would be to use small organic molecules of similar structure but variable sizes. Another option could be the use of different ideal gases—as good model systems for spherical particles with variable sizes—whose absorption into the polymer sample is driven by entropy [79] and which have relatively weak energetic interactions with the polymer matrix.

## Figures and Tables

**Table 1:** The interaction parameters employed in the present bead-spring copolymer model.

$\sigma_{\mathrm{AA}}$	$\sigma_{\mathrm{BB}}$	$\sigma_{\mathrm{SS}}$	$\sigma_{\mathrm{AB}}$	$\sigma_{\mathrm{AS}}$	$\sigma_{\mathrm{BS}}$
1	1	{0.3, 0.5, 0.8}	1	arithmetic mean	arithmetic mean
$\varepsilon_{\mathrm{AA}}$	$\varepsilon_{\mathrm{BB}}$	$\varepsilon_{\mathrm{SS}}$	$\varepsilon_{\mathrm{AB}}$	$\varepsilon_{\mathrm{AS}}$	$\varepsilon_{\mathrm{BS}}$
1	0.5	0.5	0.3	0.5	0.5

## Data Availability

The data presented in this study are available on request from the corresponding author.
